# Temporally inter-comparable maps of terrestrial wilderness and the Last of the Wild

**DOI:** 10.1038/sdata.2017.187

**Published:** 2017-12-12

**Authors:** James R. Allan, Oscar Venter, James E.M. Watson

**Affiliations:** 1 School of Earth and Environmental Sciences, University of Queensland, St Lucia, QLD 4072, Australia; 2 Natural Resource and Environmental Studies Institute, University of Northern British Columbia, Prince George, Canada 2M74Z9; 3 Wildlife Conservation Society, Global Conservation Program, Bronx, NY 10460, USA

**Keywords:** Environmental chemistry, Conservation biology

## Abstract

Wilderness areas, defined as areas free of industrial scale activities and other human pressures which result in significant biophysical disturbance, are important for biodiversity conservation and sustaining the key ecological processes underpinning planetary life-support systems. Despite their importance, wilderness areas are being rapidly eroded in extent and fragmented. Here we present the most up-to-date temporally inter-comparable maps of global terrestrial wilderness areas, which are essential for monitoring changes in their extent, and for proactively planning conservation interventions to ensure their preservation. Using maps of human pressure on the natural environment for 1993 and 2009, we identified wilderness as all ‘pressure free’ lands with a contiguous area >10,000 km^2^. These places are likely operating in a natural state and represent the most intact habitats globally. We then created a regionally representative map of wilderness following the well-established ‘Last of the Wild’ methodology; which identifies the 10% area with the lowest human pressure within each of Earth’s 60 biogeographic realms, and identifies the ten largest contiguous areas, along with all contiguous areas >10,000 km^2^.

## Background & Summary

Wilderness areas are ecologically intact landscapes free of human pressures which cause significant biophysical disturbance of the natural environment^1,2^. This includes industrial activities such as land-clearing, dense human settlements, agriculture, industry, and infrastructure development^3,4^. Importantly, this definition does not exclude indigenous peoples and communities, who have been part of wilderness areas for millennia through deep bio-cultural connections to the land^5,6^.

Natural ecological and evolutionary processes continue largely unimpeded in wilderness areas, providing a suite of high-value ecosystem services^7,8^. These include regulation of hydrological cycles at multiple scales^8–10^, and significant organic carbon stocks^11,12^. Wilderness areas are also critically important for in situ biodiversity conservation, supporting the last intact mega-faunal assemblages^3,13^, wide ranging and migratory species^14,15^, and species sensitive to exploitation by or conflicts with humans^16^. Wilderness areas are also the last remaining places on Earth where scientists can study biodiversity and natural processes free from the influence of modern society.

Maps of terrestrial wilderness areas have previously been developed by mapping the extent of a number of human pressures on the environment at both global and regional scales^3,17,18^, using the logic that the areas free of human pressure constitute ‘wilderness’. These maps have proved useful for numerous ecological and conservation analyses^18–21^. However, these maps provide a temporally static and now much outdated view of wilderness extent^7,22,23^, and there have been recent calls for a more updated product^19^.

Here we present two new data-sets of spatially and temporally intercomparable maps of global terrestrial wilderness areas for the years 1993 and 2009. We used the methodological framework outlined in the original ‘Last of the Wild’ work^17^ but utilized the recently updated ‘Human Footprint’ maps^24^. These are the most up-to-date and highest resolution globally standardized maps of cumulative human pressure on the terrestrial environment^25^. The Human Footprint is the only pressure map to have had its data validated^24^, and is widely regarded as the best available product of its kind^26^.

Our maps of wilderness areas have already been used to highlight catastrophic declines in wilderness extent over the last two decades, and show that conservation efforts has been greatly outpaced by these losses^4^. This has raised the profile of wilderness conservation globally^12,27^, and it seems that international targets for wilderness conservation may be developed shortly^12,19^. We anticipate that our maps will be important tools in the process of developing such targets, and for the conservation planning and decision making necessary to ensure representative protection of wilderness areas globally^3,28,29^.

## Methods

### The human footprint

**Figure 1:**
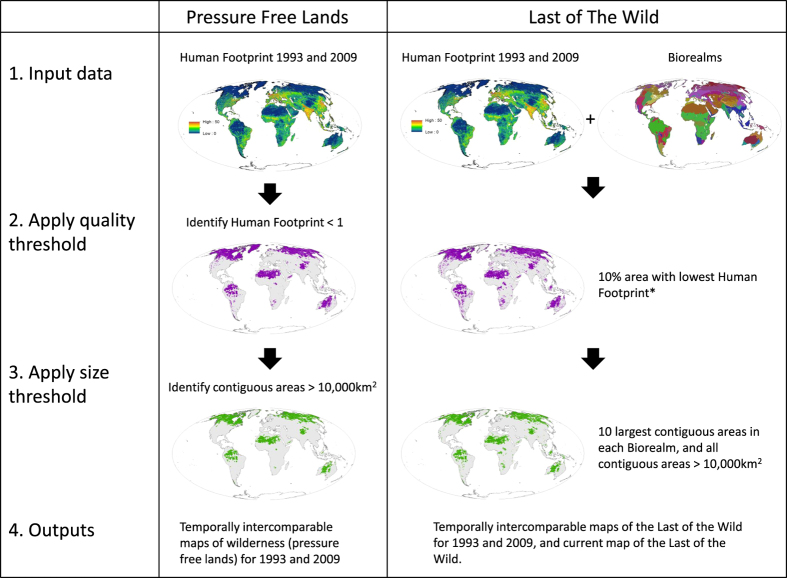
Workflow of our approach to mapping pressure free lands and the Last of the Wild. * For temporally inter-comparable maps of the Last of the Wild the 10% threshold is based on the 1993 Human Footprint for both the 1993 and 2009 maps. For the current Last of the Wild the 10% threshold is based on the 2009 Human Footprint. See methods for more detail.

To map the global extent of wilderness we utilised the recently updated Human Footprint maps for 1993 and 2009^24,25^ (Fig. 1). These are globally-standardised maps of cumulative human pressures on the terrestrial environment. At a 1 km^2^, they are the finest resolution cumulative threat maps available, as well as the most comprehensive, including data on eight human pressures globally: built environments; crop lands; pasture lands; population density; night-time lights; railways; major roadways; and navigable waterways. Following the original Human Footprint methodology^17^, individual pressures were placed within a 0–10 scale based on their estimated contribution to human pressure, and summed giving a cumulative score ranging from 0–50 for each pixel (some pressures are mutually exclusive, whilst others can co-occur). We converted the Human Footprint datasets from a continuous to an integer 0–50 scale by truncating. The integer Human Footprint datasets were used for all the analyses described in the paper. The following sections and Table 1 describe in detail how these datasets were handled to map pressure free lands and the Last of the Wild.

### Comparable maps of pressure free lands for 1993 and 2009

We created two global maps of wilderness in 1993 and 2009 by identifying all areas which are free of human pressure (Human Footprint=0), and have a contiguous area >10,000 km^2^. This size threshold has been used by others to identify wilderness areas^3,7,19^, and is consistent with the parameter values for identifying intact ecological communities in the International Union for Nature Conservation (IUCN) standards for identifying Key Biodiversity Areas^30^. Large wilderness areas separated by small areas of Human Footprint greater than ‘0’ were treated as two discreet wilderness blocks. Given the difficulty in restoring wilderness condition, locations which had a Human Footprint score >0 in 1993 but=0 in 2009 were excluded, as was Antarctica for its lack of suitable data.

### Temporally inter-comparable maps of the ‘Last of the Wild’ for 1993 and 2009

We also created global maps of the ‘Last of the Wild’ for 1993 and 2009 following the methodology developed by Sanderson *et al.*^17^. First, we created a layer of biogeographic realms (hereafter simply ‘biorealms’) as a biogeographic framework for our analysis, based on the widely used Terrestrial Ecoregions of the World^31^. The biorealms represent combinations of the world’s 14 vegetated biomes and seven biogeographic realms (for example boreal forests exist in both the Palearctic and Nearctic realms). Following established practice we excluded Antarctica and other rock and ice ecoregions^32,33^. Our resulting map contained 60 out of a possible 67 biorealms because some sub-Antarctic and Pacific islands fall beyond the extent of the Human Footprint data (Supplementary File 1).

We calculated biorealm specific thresholds on the 1993 Human Footprint scale which ensured that at least 10% of each biorealm’s land area with the lowest Human Footprint in 1993 was captured. We then selected the ten largest contiguous blocks in each biorealm and all contiguous areas >10,000 km^2^ to create the Last of the Wild dataset for 1993. The same biorealm specific thresholds identified for the 1993 map for the 10% area with the lowest Human Footprint score for 1993 were also used to map the 2009 Last of the Wild so that it is possible to directly compare changes in wilderness extent across the two time periods. Finally, we created a map of the Last of the Wild for 2009 where we calculated the biorealm specific thresholds on the 2009 Human Footprint scale which ensured that at least 10% of a biorealms land area with the lowest Human Footprint in 2009 was captured (for the previous maps we used the 1993 threshold to ensure maps from the two time periods are comparable). This map is not comparable with the 1993 map, but is important since it shows the current best quality habitat left in all the biorealms.

## Data Records

The 1 km^2^ resolution, temporally inter-comparable maps of pressure free lands and the 1993 and 2009 Last of the Wild maps [Data Citation 1] are stored in the Dryad Digital Repository where they can be accessed freely. The Dryad files can be downloaded as a single 7-zip file archive which contains an individual shapefile (.shp) for each of the five maps and excel databases containing the validation data (Table 2). The Human Footprint dataset which underpins this work is also freely available on Dryad [Data Citation 2] and contains the entire dataset for the visual validation as well as the Human Footprint maps.

## Technical Validation

The Human Footprint dataset underpinning our wilderness mapping was the first cumulative pressure map to undergo data validation^24^. High resolution satellite imagery^34^ was used to visually interpret human pressures in 3,460×1 km^2^ plots across earth’s terrestrial areas. A standard key for interpreting pressures was used and plots were also scored as certain or uncertain. Only plots where visual scores were certain (*n*=3,114) were used in the final validation exercise, and they had a median satellite imagery resolution of 0.5 meters. In general, a plot was scored as uncertain due to cloud cover or moderate resolution (15 m) imagery. The Human Footprint score for each plot was determined through overlay in ArcGIS and both the visual and Human Footprint scores were normalised to a 0–1 scale making it possible to compare the two. Comparable imagery for 1993 was not available so only the 2009 map was validated.

The pressure scores in the visual validation and the Human Footprint strongly agree. The root mean squared error (RMSE)^35^ and the Cohen kappa statistic of agreement^36^ were used to determine Human Footprint performance. The RMSE is a dimensioned (expresses average error in the units of the variable of interest) error metric for numerical predictions, and tends to heavily punish large errors. The RMSE was 0.125 on the normalised 0–1 scale indicating an average error of approximately 13%.

The Kappa statistic expresses the agreement between two categorical datasets corrected for the expected agreement, which is based on a random allocation given the relative class sizes. When calculating the kappa statistic, the 2009 Human Footprint score was considered as a match to the visual score if they were within 20% (0.2 on 0–1 scale). The Kappa statistic was 0.737 (*P*<0.01) which indicates strong agreement^36,37^. Of the visual validation plots 2,757 (88.5%) were within 20% agreement. The Human Footprint scored 94 plots 20% higher than the visual validation score and 263 of them 20% lower. This suggests that the Human Footprint may be a slightly conservative measure of pressure, mapping pressures as absent in some places where they are actually present; however, the overall agreement is strong and encouraging. The sensitivity of the Kappa statistic to different thresholds for defining agreement was tested by Venter *et al.*^24^ when validating the 2009 Human Footprint. With thresholds of within 15 and 25% the Kappa statistics were 0.565 (moderate agreement) and 0.856 (very strong agreement) respectively. This suggests some sensitivity but still shows good agreement.

**Figure 2:**
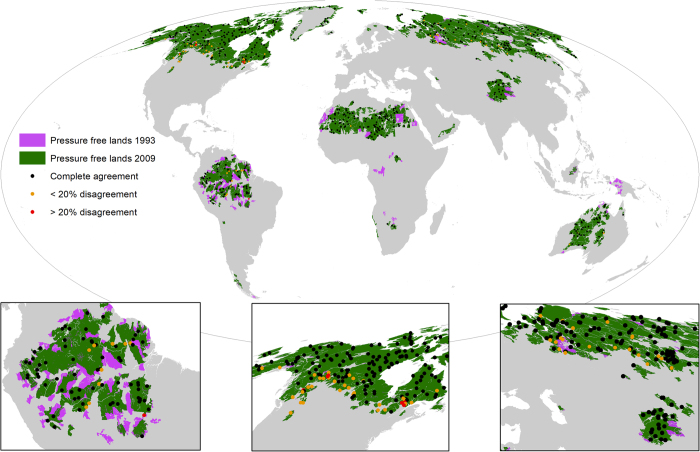
The extent of pressure free lands in 1993 (purple) and 2009 (green) with the results of the validation plots overlaid. Validation plots which were visually scored as pressure free and are therefore concordant with our definition of wilderness (Human Footprint=0) are shown in black. Validation points that disagree by<20% are shown in yellow, and those that disagree by >20% are red.

To validate our map of pressure free lands in 2009 we identified all the plots from the Human Footprint visual validation which intersect our wilderness areas and assessed if they were in fact pressure free (Fig. 2). We used 624 plots with a median imagery resolution of 2.5 meters and found that 550 (88.1%) of the plots were scored through visual interpretation as completely free of human pressure. This shows strong agreement but suggests that in some places our maps are overestimating wilderness extent. We also found that 617 (98.9%) of the plots were within 20% (0.2) agreement of a Human Footprint score of zero on the 0–1 scale (pressure free) which is encouraging, and suggests that where we do overestimate wilderness the error is relatively small.

**Figure 3:**
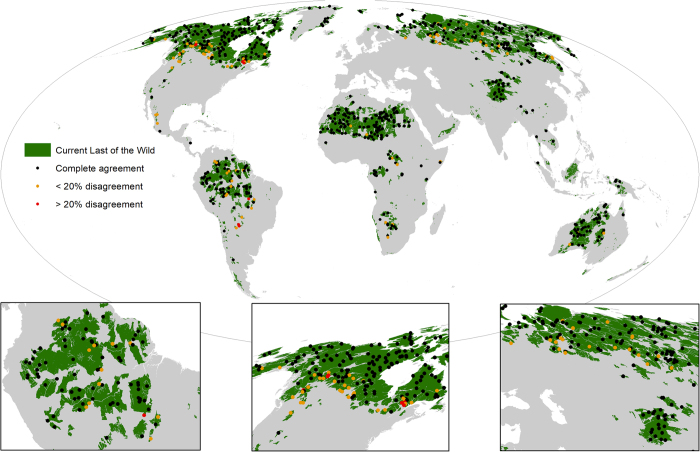
The extent of the current Last of the Wild with the results of the validation plots overlaid. Validation plots which were visually scored as pressure free and are therefore concordant with our definition of wilderness (Human Footprint=0) are shown in black. Validation points that disagree by<20% are shown in yellow, and those that disagree by >20% are red.

To validate the Last of the Wild map for 2009 we also identified all the plots from the Human Footprint visual validation which intersect those areas, and assessed if the standardised visual human pressure scores fell below standardised biorealm specific thresholds for the 10% area with the lowest Human Footprint in 2009 (Fig. 3). We used 687 plots with a median imagery resolution of 2.5 meters and found that 597 (86.9%) of the plots had visual pressure scores below their biorealm specific threshold showing strong agreement. If we consider scores up to 20% above a threshold as acceptable, then 678 (98.7%) of the plots are in agreement. Again, this suggests that the maps are overestimating wilderness in some places but that the errors are relatively small.

## Usage Notes

The maps of wilderness we present are currently the most up-to-date products available. They are temporally inter-comparable and can be used to support a range of analyses including monitoring changes in wilderness extent and fragmentation over time, and are important information for conservation planning. The maps also include essential information needed to identify areas that could potentially meet the size and intactness criteria specified in the 2016 IUCN Global Standards for identifying Key Biodiversity Areas^30^. Conserving wilderness areas is imperative for biodiversity conservation; as disturbance sensitive species disappear from human dominated landscapes, wilderness areas are becoming their last remaining strongholds^38^. These will be important sources of propagules and populations for restoration and re-wilding efforts, and serve as a baseline reference^39,40^.

Protecting wilderness areas is also important because they provide high-value ecosystem services which are being lost in human modified and degraded landscapes^8,14,41,42^. Intact functioning ecosystems sequester and protect large amounts of carbon^11^, regulate local climate regimes including hydrological cycles^43–45^, and provide a direct defence against climate related hazards such as floods, sea-level rise and cyclones^46^. Protecting intact ecosystems is humanity’s most cost effective defence against climate change^8,46^, and may also prove to be the most cost effective way of meeting many of the United Nations Sustainable Development Goals (SDG’s)^47,48^. The protection of wilderness areas could also serve as a direct indicator for progress towards certain SDG’s, such as goal 15 which relates to biodiversity and ecosystem conservation^47^.

Many of the ecosystem services derived from wilderness areas are a direct result of their size, which allows them to act as complete self-organising systems^17^. This has important implications for their conservation since damage in one area can affect the function of the entire system^49^. For example, it is estimated that the Amazon needs 60% of its forest cover to retain its hydrological cycle^50^. We anticipate our maps will be important tools for identifying places where conservation actions must occur at the ecosystem scale, and can help guide conservation efforts such as the implementation of mega-reserves^49^.

The maps of wilderness we present have several important differences to other recently published products such as maps of intact forest landscapes (IFL’s)^51^. IFL’s are satellite derived maps of the ecological state of the environment, whilst our wilderness maps are derived from maps of pressures or ‘threats’. Pressures are actions which have the potential to damage nature, and therefore can drive changes in the ecological state of a system^52^. Cumulative pressure maps such as the Human Footprint also combine top-down remotely sensed data and bottom up survey data to surmount the limitations of remotely sensed data such as lower accuracy in arid environments^25,53,54^. Most importantly, our maps are not limited to a particular biome (e.g., forests), but rather span and consistently represent all non-Antarctic land areas.

Our work is subject to several caveats worthy of discussion. The Human Footprint relies on datasets which are globally comparable, but in some areas may not have the full extent of infrastructure that national or sub-national datasets contain, or reflect all the pressures which could potentially impact on the wilderness quality of an area. For example, threats such as poaching, logging, invasive species, pollution and climate change are not directly captured, although many of them are often highly correlated to the pressures that were included in the Human Footprint^24,25^, such as human population density and road networks. There is a risk that the Human Footprint sometimes maps pressures as absent where they are actually present, underestimating human pressure in those parts of the world. This in turn suggests that our maps of wilderness are likely overestimates, and would benefit from being downscaled when used in a national or sub-national context^55^.

## Additional information

**How to cite this article:** Allan, J. R. *et al.* Temporally inter-comparable maps of terrestrial wilderness and the Last of the Wild. *Sci. Data* 4:170187 doi: 10.1038/sdata.2017.187 (2017).

**Publisher’s note:** Springer Nature remains neutral with regard to jurisdictional claims in published maps and institutional affiliations.

## Supplementary Material



Supplementary File 1

## Figures and Tables

**Table 1:** Summary of data inputs, manipulations and outputs in the wilderness and Last of the Wild workflow.

**Source**	**Data used**	**Temporal range**	**Resolution**	**Data manipulations**	**Outputs**
Data Citation 1	Human Footprint maps	1993–2009	1 km^2^	1) Reclassify raster to identify all cells with Human Footprint =02) Convert to shapefile (.shp) and calculate polygon areas3) Identify all contiguous areas >10,000 km^2^4) These are pressure free lands (wilderness)	Pressure_free_lands_1993.shpPressure_free_lands_2009.shp
REF 31	Map of terrestrial ecoregions of the world	NA	NA	1) Intersect Biomes with Biogeographic Realms to create ‘Biorealms’2) This is the biogeographical framework for identifying the Last of the Wild	Biorealms.shp
Data Citation 2	Human Footprint maps	1993–2009	1 km^2^	1) Tabulate area of each Human Footprint score in each biorealm using 1993 Human Footprint2) Identify threshold for the 10% area with lowest Human Footprint in each biorealm3) Identify area within each biorealm which falls below this threshold using A) 1993 Human Footprint and B) 2009 Human Footprint4) Convert to shapefile (.shp) and calculate polygon areas5) Identify the 10 largest contiguous areas in each biorealm and all areas >10,000 km^2^ for A and B	LoW_1993.shpLoW_2009_comparable.shp
Data Citation 2	Human Footprint maps	2009	1 km^2^	1) Tabulate area of each Human Footprint score in each biorealm using 2009 Human Footprint2) Identify threshold for the 10% area with lowest Human Footprint in each biorealm3) Identify area within each biorealms which falls below this threshold using 2009 Human Footprint4) Convert to shapefile (.shp) and calculate polygon areas5) Identify the 10 largest contiguous areas in each biorealm and all areas >10,000 km^2^	LoW_2009_current.shp

**Table 2:** The name, description and type of data included in the Wilderness_maps.zip file.

**Name**	**Description**	**Format**
Pressure_free_lands_93	Temporally comparable map of pressure free lands for 1993	Shapefile
Pressure_free_lands_09	Temporally comparable map of pressure free lands for 2009	Shapefile
LoW_1993	Temporally comparable map of the Last of the Wild for 1993	Shapefile
LoW_2009_comparable	Temporally comparable map of the Last of the Wild for 2009	Shapefile
LoW_2009_current	Map of the current last of the Wild (2009 but not temporally comparable)	Shapefile
Validation_last_of_the_wild_2009_current	Excel database with the visual validation points for the current Last of the Wild Map	Microsoft Office Excel Worksheet
Validation_pressure_free_lands_2009	Excel database with the visual validation points for pressure free lands in 2009	Microsoft Office Excel Worksheet
