# Supplementary material for: Temporally inter-comparable maps of terrestrial wilderness and the Last of the Wild
Source: Sci Data. 2017 Dec 12;4:170187. doi: 10.1038/sdata.2017.187 (PMC5726312; doi:10.1038/sdata.2017.187)
Supplement: Supplementary File 1 [file sdata2017187-s2.docx]

**Supplementary Information for “Temporally inter-comparable maps of terrestrial wilderness and the Last of the Wild”**

**James R. Allan, Oscar Venter, James E.M. Watson**

corresponding author: James Allan (j.allan2@uq.edu.au)

**Contents:**

**Page 2: Supplementary Table 1. *Key area and threshold statistics for The Last of the Wild 1993***

**Page 6: Supplementary Table 2. *Key area and threshold statistics for the Last of the Wild 2009 (temporally inter-comparable)***

**Page 11: Supplementary Table 3. *Key area and threshold statistics for the current Last of the Wild 2009 (not temporally inter-comparable)***

**Page 15: Supplementary Table 4. *Human Footprint statistics for 2009 within biorealms***

| ***Supplementary Table 1. Key area and threshold statistics for The Last of the Wild 1993*** | | | | | | | | |
| --- | --- | --- | --- | --- | --- | --- | --- | --- |
| **Biorealm** | **Realm** | **Biome** | **Area of biorealm** | **Human Footprint threshold 1993** | **Land area below Human Footprint threshold 1993 (km2)** | **Land area below Human Footprint threshold 1993 (%)** | **Last of the Wild 1993 (km2)** | **Last of the Wild 1993 (%)** |
| 1 AA | Australasia | Tropical and subtropical moist broadleaf forests | 1118906.7 | 0 | 169647.7 | 15.2 | 136722.7 | 12.2 |
| 1 AT | Afrotropic | Tropical and subtropical moist broadleaf forests | 3476862.7 | 1 | 529207.0 | 15.2 | 245183.1 | 7.1 |
| 1 IM | Indo-Malay | Tropical and subtropical moist broadleaf forests | 5356337.8 | 2 | 563964.1 | 10.5 | 362096.4 | 6.8 |
| 1 NT | Neotropic | Tropical and subtropical moist broadleaf forests | 9253107.9 | 0 | 3902447.7 | 42.2 | 3670281.0 | 39.7 |
| 1 PA | Palearctic | Tropical and subtropical moist broadleaf forests | 511013.2 | 6 | 54136.3 | 10.6 | 21128.2 | 4.1 |
| 10 AA | Australasia | Montane grasslands and savannas | 67647.5 | 0 | 10593.4 | 15.7 | 7827.0 | 11.6 |
| 10 AT | Afrotropic | Montane grasslands and savannas | 863697.7 | 4 | 104304.4 | 12.1 | 23028.9 | 2.7 |
| 10 IM | Indo-Malay | Montane grasslands and savannas | 4348.6 | 3 | 505.0 | 11.6 | 480.1 | 11.0 |
| 10 NT | Neotropic | Montane grasslands and savannas | 873700.6 | 0 | 216686.0 | 24.8 | 54736.4 | 6.3 |
| 10 PA | Palearctic | Montane grasslands and savannas | 3392417.1 | 0 | 864438.0 | 25.5 | 731527.3 | 21.6 |
| 11 NA | Nearctic | Tundra | 4067214.1 | 0 | 3951046.4 | 97.1 | 3574517.0 | 87.9 |
| 11 PA | Palearctic | Tundra | 3937806.6 | 0 | 3398978.9 | 86.3 | 2805248.6 | 71.2 |
| 12 AA | Australasia | Mediterranean forests woodlands and scrub | 800729.8 | 0 | 171961.2 | 21.5 | 78671.8 | 9.8 |
| 12 AT | Afrotropic | Mediterranean forests woodlands and scrub | 95271.7 | 3 | 18829.0 | 19.8 | 5887.8 | 6.2 |
| 12 NA | Nearctic | Mediterranean forests woodlands and scrub | 119336.2 | 1 | 15612.7 | 13.1 | 10501.4 | 8.8 |
| 12 NT | Neotropic | Mediterranean forests woodlands and scrub | 147628.7 | 2 | 30331.4 | 20.5 | 10504.1 | 7.1 |
| 12 PA | Palearctic | Mediterranean forests woodlands and scrub | 2022502.5 | 4 | 305676.3 | 15.1 | 27017.0 | 1.3 |
| 13 AA | Australasia | Deserts and xeric shrublands | 3577947.0 | 0 | 1969457.1 | 55.0 | 1584482.1 | 44.3 |
| 13 AT | Afrotropic | Deserts and xeric shrublands | 2391452.7 | 1 | 446988.4 | 18.7 | 133907.5 | 5.6 |
| 13 IM | Indo-Malay | Deserts and xeric shrublands | 1086691.9 | 5 | 117444.8 | 10.8 | 42041.4 | 3.9 |
| 13 NA | Nearctic | Deserts and xeric shrublands | 2320649.2 | 1 | 288268.8 | 12.4 | 68391.8 | 2.9 |
| 13 NT | Neotropic | Deserts and xeric shrublands | 1155761.4 | 2 | 212708.2 | 18.4 | 28635.4 | 2.5 |
| 13 PA | Palearctic | Deserts and xeric shrublands | 17359489.8 | 0 | 6405858.5 | 36.9 | 5064135.0 | 29.2 |
| 14 AA | Australasia | Mangrove | 22735.4 | 0 | 9539.4 | 42.0 | 7506.0 | 33.0 |
| 14 AT | Afrotropic | Mangrove | 70874.6 | 3 | 10005.1 | 14.1 | 6219.8 | 8.8 |
| 14 IM | Indo-Malay | Mangrove | 96201.6 | 6 | 25234.0 | 26.2 | 6148.0 | 6.4 |
| 14 NT | Neotropic | Mangrove | 101193.3 | 2 | 18556.9 | 18.3 | 8017.2 | 7.9 |
| 2 AA | Australasia | Tropical and subtropical dry broadleaf forests | 82421.0 | 3 | 9031.4 | 11.0 | 6726.8 | 8.2 |
| 2 AT | Afrotropic | Tropical and subtropical dry broadleaf forests | 192019.4 | 2 | 35616.4 | 18.5 | 15215.0 | 7.9 |
| 2 IM | Indo-Malay | Tropical and subtropical dry broadleaf forests | 1529020.2 | 6 | 230349.5 | 15.1 | 70167.2 | 4.6 |
| 2 NA | Nearctic | Tropical and subtropical dry broadleaf forests | 50750.2 | 3 | 10930.6 | 21.5 | 4818.1 | 9.5 |
| 2 NT | Neotropic | Tropical and subtropical dry broadleaf forests | 1136971.8 | 1 | 137222.5 | 12.1 | 67489.5 | 5.9 |
| 3 IM | Indo-Malay | Tropical and subtropical coniferous forests | 95953.6 | 6 | 23049.6 | 24.0 | 6922.2 | 7.2 |
| 3 NA | Nearctic | Tropical and subtropical coniferous forests | 289014.2 | 2 | 88193.2 | 30.5 | 32530.5 | 11.3 |
| 3 NT | Neotropic | Tropical and subtropical coniferous forests | 323971.9 | 3 | 38505.1 | 11.9 | 15572.4 | 4.8 |
| 4 AA | Australasia | Temperate broadleaf and mixed forests | 718365.7 | 1 | 152718.3 | 21.3 | 42208.7 | 5.9 |
| 4 IM | Indo-Malay | Temperate broadleaf and mixed forests | 149970.5 | 2 | 22775.6 | 15.2 | 12740.5 | 8.5 |
| 4 NA | Nearctic | Temperate broadleaf and mixed forests | 2820912.1 | 0 | 308454.9 | 10.9 | 179615.9 | 6.4 |
| 4 NT | Neotropic | Temperate broadleaf and mixed forests | 380188.1 | 0 | 109717.5 | 28.9 | 83372.9 | 21.9 |
| 4 PA | Palearctic | Temperate broadleaf and mixed forests | 8594566.1 | 4 | 1040425.3 | 12.1 | 206300.8 | 2.4 |
| 5 IM | Indo-Malay | Temperate coniferous forests | 67304.2 | 2 | 8361.2 | 12.4 | 6831.0 | 10.1 |
| 5 NA | Nearctic | Temperate coniferous forests | 2269320.3 | 0 | 824187.2 | 36.3 | 440744.1 | 19.4 |
| 5 PA | Palearctic | Temperate coniferous forests | 1705316.2 | 0 | 153931.4 | 9.0 | 126504.3 | 7.4 |
| 6 NA | Nearctic | Boreal forests taiga | 5065333.8 | 0 | 4676396.7 | 92.3 | 4315099.0 | 85.2 |
| 6 PA | Palearctic | Boreal forests taiga | 9930242.1 | 0 | 5682187.9 | 57.2 | 4770608.3 | 48.0 |
| 7 AA | Australasia | Tropical and subtropical grasslands savannas | 2158470.5 | 0 | 769708.8 | 35.7 | 375606.8 | 17.4 |
| 7 AT | Afrotropic | Tropical and subtropical grasslands savannas | 13984546.5 | 1 | 1718950.6 | 12.3 | 809573.6 | 5.8 |
| 7 IM | Indo-Malay | Tropical and subtropical grasslands savannas | 34648.8 | 8 | 4507.2 | 13.0 | 1389.0 | 4.0 |
| 7 NA | Nearctic | Tropical and subtropical grasslands savannas | 73980.5 | 3 | 9933.5 | 13.4 | 4098.4 | 5.5 |
| 7 NT | Neotropic | Tropical and subtropical grasslands savannas | 3991391.5 | 1 | 956366.6 | 24.0 | 262608.1 | 6.6 |
| 7 OC | Oceania | Tropical and subtropical grasslands savannas | 2910.1 | 1 | 1122.5 | 38.6 | 1121.7 | 38.5 |
| 8 AA | Australasia | Temperate grasslands savannas and shrublands | 628829.0 | 2 | 112537.1 | 17.9 | 10697.8 | 1.7 |
| 8 AT | Afrotropic | Temperate grasslands savannas and shrublands | 25604.6 | 1 | 4245.3 | 16.6 | 4142.3 | 16.2 |
| 8 NA | Nearctic | Temperate grasslands savannas and shrublands | 3096634.6 | 1 | 482931.5 | 15.6 | 48400.3 | 1.6 |
| 8 NT | Neotropic | Temperate grasslands savannas and shrublands | 1623813.7 | 1 | 198135.3 | 12.2 | 40253.9 | 2.5 |
| 8 PA | Palearctic | Temperate grasslands savannas and shrublands | 4700423.2 | 3 | 870101.8 | 18.5 | 95074.1 | 2.0 |
| 9 AT | Afrotropic | Flooded grasslands and savannas | 446169.0 | 2 | 65504.5 | 14.7 | 37775.4 | 8.5 |
| 9 IM | Indo-Malay | Flooded grasslands and savannas | 27301.5 | 0 | 7445.2 | 27.3 | 6971.0 | 25.5 |
| 9 NT | Neotropic | Flooded grasslands and savannas | 270063.2 | 1 | 62177.8 | 23.0 | 32597.1 | 12.1 |
| 9 PA | Palearctic | Flooded grasslands and savannas | 332514.4 | 0 | 38168.4 | 11.5 | 23204.7 | 7.0 |

| ***Supplementary Table 2. Key area and threshold statistics for the Last of the Wild 2009 (temporally inter-comparable)*** | | | | | | | | |
| --- | --- | --- | --- | --- | --- | --- | --- | --- |
| **Biorealm** | **Realm** | **Biome** | **Area of biorealm** | **Human Footprint threshold 1993** | **Land area below Human Footprint threshold 2009 (km2)** | **Land area below Human Footprint threshold 2009 (%)** | **Last of the Wild 2009 Comparable (km2)** | **Last of the Wild Comparable 2009 (%)** |
| 1 AA | Australasia | Tropical and subtropical moist broadleaf forests | 1118906.7 | 0 | 83294.8 | 7.4 | 47257.4 | 4.2 |
| 1 AT | Afrotropic | Tropical and subtropical moist broadleaf forests | 3476862.7 | 1 | 323576.2 | 9.3 | 184485.3 | 5.3 |
| 1 IM | Indo-Malay | Tropical and subtropical moist broadleaf forests | 5356337.8 | 2 | 403279.7 | 7.5 | 261355.1 | 4.9 |
| 1 NT | Neotropic | Tropical and subtropical moist broadleaf forests | 9253107.9 | 0 | 3117239.2 | 33.7 | 2883375.2 | 31.2 |
| 1 PA | Palearctic | Tropical and subtropical moist broadleaf forests | 511013.2 | 6 | 35598.6 | 7.0 | 14419.4 | 2.8 |
| 10 AA | Australasia | Montane grasslands and savannas | 67647.5 | 0 | 8579.1 | 12.7 | 6154.3 | 9.1 |
| 10 AT | Afrotropic | Montane grasslands and savannas | 863697.7 | 4 | 88960.9 | 10.3 | 20465.2 | 2.4 |
| 10 IM | Indo-Malay | Montane grasslands and savannas | 4348.6 | 3 | 0.0 | 0.0 | 0.0 | 0.0 |
| 10 NT | Neotropic | Montane grasslands and savannas | 873700.6 | 0 | 182375.8 | 20.9 | 49269.5 | 5.6 |
| 10 PA | Palearctic | Montane grasslands and savannas | 3392417.1 | 0 | 770993.5 | 22.7 | 657639.3 | 19.4 |
| 11 NA | Nearctic | Tundra | 4067214.1 | 0 | 3934793.2 | 96.7 | 3609620.8 | 88.7 |
| 11 PA | Palearctic | Tundra | 3937806.6 | 0 | 3418060.4 | 86.8 | 2786548.2 | 70.8 |
| 12 AA | Australasia | Mediterranean forests woodlands and scrub | 800729.8 | 0 | 162717.4 | 20.3 | 76297.8 | 9.5 |
| 12 AT | Afrotropic | Mediterranean forests woodlands and scrub | 95271.7 | 3 | 17109.4 | 18.0 | 5502.4 | 5.8 |
| 12 NA | Nearctic | Mediterranean forests woodlands and scrub | 119336.2 | 1 | 12455.9 | 10.4 | 8767.3 | 7.3 |
| 12 NT | Neotropic | Mediterranean forests woodlands and scrub | 147628.7 | 2 | 22289.6 | 15.1 | 8119.1 | 5.5 |
| 12 PA | Palearctic | Mediterranean forests woodlands and scrub | 2022502.5 | 4 | 268305.7 | 13.3 | 26475.1 | 1.3 |
| 13 AA | Australasia | Deserts and xeric shrublands | 3577947.0 | 0 | 1969453.8 | 55.0 | 1584525.1 | 44.3 |
| 13 AT | Afrotropic | Deserts and xeric shrublands | 2391452.7 | 1 | 406137.0 | 17.0 | 135061.9 | 5.6 |
| 13 IM | Indo-Malay | Deserts and xeric shrublands | 1086691.9 | 5 | 67051.8 | 6.2 | 25727.1 | 2.4 |
| 13 NA | Nearctic | Deserts and xeric shrublands | 2320649.2 | 1 | 265053.6 | 11.4 | 68266.1 | 2.9 |
| 13 NT | Neotropic | Deserts and xeric shrublands | 1155761.4 | 2 | 169984.2 | 14.7 | 23862.9 | 2.1 |
| 13 PA | Palearctic | Deserts and xeric shrublands | 17359489.8 | 0 | 5846807.6 | 33.7 | 4601151.8 | 26.5 |
| 14 AA | Australasia | Mangrove | 22735.4 | 0 | 5027.7 | 22.1 | 3912.0 | 17.2 |
| 14 AT | Afrotropic | Mangrove | 70874.6 | 3 | 5181.8 | 7.3 | 3319.0 | 4.7 |
| 14 IM | Indo-Malay | Mangrove | 96201.6 | 6 | 3821.0 | 4.0 | 1678.0 | 1.7 |
| 14 NT | Neotropic | Mangrove | 101193.3 | 2 | 16298.7 | 16.1 | 7436.6 | 7.3 |
| 2 AA | Australasia | Tropical and subtropical dry broadleaf forests | 82421.0 | 3 | 3482.9 | 4.2 | 2448.0 | 3.0 |
| 2 AT | Afrotropic | Tropical and subtropical dry broadleaf forests | 192019.4 | 2 | 21990.2 | 11.5 | 11071.5 | 5.8 |
| 2 IM | Indo-Malay | Tropical and subtropical dry broadleaf forests | 1529020.2 | 6 | 162076.1 | 10.6 | 60111.7 | 3.9 |
| 2 NA | Nearctic | Tropical and subtropical dry broadleaf forests | 50750.2 | 3 | 12208.1 | 24.1 | 5369.7 | 10.6 |
| 2 NT | Neotropic | Tropical and subtropical dry broadleaf forests | 1136971.8 | 1 | 90293.6 | 7.9 | 46799.5 | 4.1 |
| 3 IM | Indo-Malay | Tropical and subtropical coniferous forests | 95953.6 | 6 | 11858.5 | 12.4 | 5499.2 | 5.7 |
| 3 NA | Nearctic | Tropical and subtropical coniferous forests | 289014.2 | 2 | 84656.2 | 29.3 | 31467.3 | 10.9 |
| 3 NT | Neotropic | Tropical and subtropical coniferous forests | 323971.9 | 3 | 40201.7 | 12.4 | 14668.4 | 4.5 |
| 4 AA | Australasia | Temperate broadleaf and mixed forests | 718365.7 | 1 | 135860.7 | 18.9 | 38822.3 | 5.4 |
| 4 IM | Indo-Malay | Temperate broadleaf and mixed forests | 149970.5 | 2 | 16909.8 | 11.3 | 10352.4 | 6.9 |
| 4 NA | Nearctic | Temperate broadleaf and mixed forests | 2820912.1 | 0 | 305516.7 | 10.8 | 178537.0 | 6.3 |
| 4 NT | Neotropic | Temperate broadleaf and mixed forests | 380188.1 | 0 | 98723.8 | 26.0 | 77311.7 | 20.3 |
| 4 PA | Palearctic | Temperate broadleaf and mixed forests | 8594566.1 | 4 | 1049391.3 | 12.2 | 221845.5 | 2.6 |
| 5 IM | Indo-Malay | Temperate coniferous forests | 67304.2 | 2 | 7418.1 | 11.0 | 6287.8 | 9.3 |
| 5 NA | Nearctic | Temperate coniferous forests | 2269320.3 | 0 | 776525.4 | 34.2 | 428162.0 | 18.9 |
| 5 PA | Palearctic | Temperate coniferous forests | 1705316.2 | 0 | 163604.1 | 9.6 | 132725.9 | 7.8 |
| 6 NA | Nearctic | Boreal forests taiga | 5065333.8 | 0 | 4674256.9 | 92.3 | 4311465.6 | 85.1 |
| 6 PA | Palearctic | Boreal forests taiga | 9930242.1 | 0 | 6024698.0 | 60.7 | 4931674.9 | 49.7 |
| 7 AA | Australasia | Tropical and subtropical grasslands savannas | 2158470.5 | 0 | 761154.9 | 35.3 | 356661.3 | 16.5 |
| 7 AT | Afrotropic | Tropical and subtropical grasslands savannas | 13984546.5 | 1 | 1264135.5 | 9.0 | 639414.0 | 4.6 |
| 7 IM | Indo-Malay | Tropical and subtropical grasslands savannas | 34648.8 | 8 | 1287.6 | 3.7 | 522.8 | 1.5 |
| 7 NA | Nearctic | Tropical and subtropical grasslands savannas | 73980.5 | 3 | 19089.8 | 25.8 | 10334.3 | 14.0 |
| 7 NT | Neotropic | Tropical and subtropical grasslands savannas | 3991391.5 | 1 | 772256.2 | 19.3 | 183414.3 | 4.6 |
| 7 OC | Oceania | Tropical and subtropical grasslands savannas | 2910.1 | 1 | 962.0 | 33.1 | 960.9 | 33.0 |
| 8 AA | Australasia | Temperate grasslands savannas and shrublands | 628829.0 | 2 | 85412.1 | 13.6 | 9783.1 | 1.6 |
| 8 AT | Afrotropic | Temperate grasslands savannas and shrublands | 25604.6 | 1 | 681.2 | 2.7 | 681.2 | 2.7 |
| 8 NA | Nearctic | Temperate grasslands savannas and shrublands | 3096634.6 | 1 | 590558.6 | 19.1 | 50872.7 | 1.6 |
| 8 NT | Neotropic | Temperate grasslands savannas and shrublands | 1623813.7 | 1 | 181811.0 | 11.2 | 36623.9 | 2.3 |
| 8 PA | Palearctic | Temperate grasslands savannas and shrublands | 4700423.2 | 3 | 710088.8 | 15.1 | 82133.5 | 1.7 |
| 9 AT | Afrotropic | Flooded grasslands and savannas | 446169.0 | 2 | 45801.3 | 10.3 | 33655.6 | 7.5 |
| 9 IM | Indo-Malay | Flooded grasslands and savannas | 27301.5 | 0 | 11202.4 | 41.0 | 10014.2 | 36.7 |
| 9 NT | Neotropic | Flooded grasslands and savannas | 270063.2 | 1 | 54468.3 | 20.2 | 26831.4 | 9.9 |
| 9 PA | Palearctic | Flooded grasslands and savannas | 332514.4 | 0 | 35976.7 | 10.8 | 21479.4 | 6.5 |

| ***Supplementary Table 3. Key area and threshold statistics for the current Last of the Wild 2009 (not temporally inter-comparable)*** | | | | | | | | |
| --- | --- | --- | --- | --- | --- | --- | --- | --- |
| **Biorealm** | **Realm** | **Biome** | **Area of biorealm** | **Human Footprint threshold 1993** | **Land area below Human Footprint threshold 2009 (km2)** | **Land area below Human Footprint threshold 2009 (%)** | **Last of the Wild 2009 (km2)** | **Last of the Wild 2009 (%)** |
| 1 AA | Australasia | Tropical and subtropical moist broadleaf forests | 1118906.7 | 1 | 228628.9 | 20.4 | 153628.9 | 13.7 |
| 1 AT | Afrotropic | Tropical and subtropical moist broadleaf forests | 3476862.7 | 2 | 605085.6 | 17.4 | 265794.6 | 7.6 |
| 1 IM | Indo-Malay | Tropical and subtropical moist broadleaf forests | 5356337.8 | 3 | 631132.7 | 11.8 | 322648.7 | 6.0 |
| 1 NT | Neotropic | Tropical and subtropical moist broadleaf forests | 9253107.9 | 0 | 3117219.9 | 33.7 | 2883375.2 | 31.2 |
| 1 PA | Palearctic | Tropical and subtropical moist broadleaf forests | 511013.2 | 7 | 88175.0 | 17.3 | 21903.7 | 4.3 |
| 10 AA | Australasia | Montane grasslands and savannas | 67647.5 | 0 | 8623.0 | 12.7 | 6158.6 | 9.1 |
| 10 AT | Afrotropic | Montane grasslands and savannas | 863697.7 | 4 | 89030.4 | 10.3 | 20470.8 | 2.4 |
| 10 IM | Indo-Malay | Montane grasslands and savannas | 4348.6 | 4 | 863.3 | 19.9 | 843.2 | 19.4 |
| 10 NT | Neotropic | Montane grasslands and savannas | 873700.6 | 0 | 182376.0 | 20.9 | 49269.5 | 5.6 |
| 10 PA | Palearctic | Montane grasslands and savannas | 3392417.1 | 0 | 770994.0 | 22.7 | 657639.3 | 19.4 |
| 11 NA | Nearctic | Tundra | 4067214.1 | 0 | 3934793.4 | 96.7 | 3569443.2 | 87.8 |
| 11 PA | Palearctic | Tundra | 3937806.6 | 0 | 3418060.8 | 86.8 | 2786549.0 | 70.8 |
| 12 AA | Australasia | Mediterranean forests woodlands and scrub | 800729.8 | 0 | 162717.4 | 20.3 | 76297.8 | 9.5 |
| 12 AT | Afrotropic | Mediterranean forests woodlands and scrub | 95271.7 | 3 | 17120.8 | 18.0 | 5503.1 | 5.8 |
| 12 NA | Nearctic | Mediterranean forests woodlands and scrub | 119336.2 | 1 | 12455.9 | 10.4 | 8767.2 | 7.3 |
| 12 NT | Neotropic | Mediterranean forests woodlands and scrub | 147628.7 | 2 | 22289.5 | 15.1 | 8119.1 | 5.5 |
| 12 PA | Palearctic | Mediterranean forests woodlands and scrub | 2022502.5 | 4 | 268305.0 | 13.3 | 26475.1 | 1.3 |
| 13 AA | Australasia | Deserts and xeric shrublands | 3577947.0 | 0 | 1969453.7 | 55.0 | 1584525.1 | 44.3 |
| 13 AT | Afrotropic | Deserts and xeric shrublands | 2391452.7 | 1 | 406475.8 | 17.0 | 135066.8 | 5.6 |
| 13 IM | Indo-Malay | Deserts and xeric shrublands | 1086691.9 | 6 | 134025.0 | 12.3 | 44016.5 | 4.1 |
| 13 NA | Nearctic | Deserts and xeric shrublands | 2320649.2 | 1 | 264997.9 | 11.4 | 68266.1 | 2.9 |
| 13 NT | Neotropic | Deserts and xeric shrublands | 1155761.4 | 2 | 169984.1 | 14.7 | 23862.9 | 2.1 |
| 13 PA | Palearctic | Deserts and xeric shrublands | 17359489.8 | 0 | 5847098.1 | 33.7 | 4601164.0 | 26.5 |
| 14 AA | Australasia | Mangrove | 22735.4 | 0 | 5163.5 | 22.7 | 3913.7 | 17.2 |
| 14 AT | Afrotropic | Mangrove | 70874.6 | 4 | 11501.9 | 16.2 | 5480.0 | 7.7 |
| 14 IM | Indo-Malay | Mangrove | 96201.6 | 7 | 26395.0 | 27.4 | 8195.0 | 8.5 |
| 14 NT | Neotropic | Mangrove | 101193.3 | 2 | 16294.1 | 16.1 | 7436.6 | 7.3 |
| 2 AA | Australasia | Tropical and subtropical dry broadleaf forests | 82421.0 | 6 | 9286.3 | 11.3 | 5220.7 | 6.3 |
| 2 AT | Afrotropic | Tropical and subtropical dry broadleaf forests | 192019.4 | 2 | 22149.3 | 11.5 | 11121.1 | 5.8 |
| 2 IM | Indo-Malay | Tropical and subtropical dry broadleaf forests | 1529020.2 | 6 | 162162.7 | 10.6 | 60137.8 | 3.9 |
| 2 NA | Nearctic | Tropical and subtropical dry broadleaf forests | 50750.2 | 2 | 5285.6 | 10.4 | 3207.5 | 6.3 |
| 2 NT | Neotropic | Tropical and subtropical dry broadleaf forests | 1136971.8 | 1 | 90289.2 | 7.9 | 46799.5 | 4.1 |
| 3 IM | Indo-Malay | Tropical and subtropical coniferous forests | 95953.6 | 7 | 24197.1 | 25.2 | 6684.5 | 7.0 |
| 3 NA | Nearctic | Tropical and subtropical coniferous forests | 289014.2 | 2 | 84539.9 | 29.3 | 31467.3 | 10.9 |
| 3 NT | Neotropic | Tropical and subtropical coniferous forests | 323971.9 | 3 | 40201.7 | 12.4 | 14668.4 | 4.5 |
| 4 AA | Australasia | Temperate broadleaf and mixed forests | 718365.7 | 1 | 135860.8 | 18.9 | 38822.3 | 5.4 |
| 4 IM | Indo-Malay | Temperate broadleaf and mixed forests | 149970.5 | 2 | 17030.2 | 11.4 | 10356.9 | 6.9 |
| 4 NA | Nearctic | Temperate broadleaf and mixed forests | 2820912.1 | 0 | 305488.8 | 10.8 | 178537.0 | 6.3 |
| 4 NT | Neotropic | Temperate broadleaf and mixed forests | 380188.1 | 0 | 98722.8 | 26.0 | 77311.5 | 20.3 |
| 4 PA | Palearctic | Temperate broadleaf and mixed forests | 8594566.1 | 2 | 1049428.3 | 12.2 | 221850.6 | 2.6 |
| 5 IM | Indo-Malay | Temperate coniferous forests | 67304.2 | 2 | 7524.1 | 11.2 | 6287.8 | 9.3 |
| 5 NA | Nearctic | Temperate coniferous forests | 2269320.3 | 0 | 776516.2 | 34.2 | 428162.0 | 18.9 |
| 5 PA | Palearctic | Temperate coniferous forests | 1705316.2 | 0 | 163642.9 | 9.6 | 132726.1 | 7.8 |
| 6 NA | Nearctic | Boreal forests taiga | 5065333.8 | 0 | 4674258.7 | 92.3 | 4311465.9 | 85.1 |
| 6 PA | Palearctic | Boreal forests taiga | 9930242.1 | 0 | 6024696.9 | 60.7 | 4941674.4 | 49.8 |
| 7 AA | Australasia | Tropical and subtropical grasslands savannas | 2158470.5 | 0 | 761238.8 | 35.3 | 356661.6 | 16.5 |
| 7 AT | Afrotropic | Tropical and subtropical grasslands savannas | 13984546.5 | 2 | 2324803.6 | 16.6 | 1030973.3 | 7.4 |
| 7 IM | Indo-Malay | Tropical and subtropical grasslands savannas | 34648.8 | 12 | 3525.1 | 10.2 | 862.9 | 2.5 |
| 7 NA | Nearctic | Tropical and subtropical grasslands savannas | 73980.5 | 2 | 9507.8 | 12.9 | 7224.8 | 9.8 |
| 7 NT | Neotropic | Tropical and subtropical grasslands savannas | 3991391.5 | 1 | 772257.4 | 19.3 | 183414.3 | 4.6 |
| 7 OC | Oceania | Tropical and subtropical grasslands savannas | 2910.1 | 1 | 962.0 | 33.1 | 960.9 | 33.0 |
| 8 AA | Australasia | Temperate grasslands savannas and shrublands | 628829.0 | 2 | 85412.1 | 13.6 | 9783.1 | 1.6 |
| 8 AT | Afrotropic | Temperate grasslands savannas and shrublands | 25604.6 | 2 | 4148.4 | 16.2 | 3997.5 | 15.6 |
| 8 NA | Nearctic | Temperate grasslands savannas and shrublands | 3096634.6 | 1 | 590558.7 | 19.1 | 50872.7 | 1.6 |
| 8 NT | Neotropic | Temperate grasslands savannas and shrublands | 1623813.7 | 1 | 181810.0 | 11.2 | 36624.2 | 2.3 |
| 8 PA | Palearctic | Temperate grasslands savannas and shrublands | 4700423.2 | 3 | 710088.8 | 15.1 | 82133.7 | 1.7 |
| 9 AT | Afrotropic | Flooded grasslands and savannas | 446169.0 | 2 | 45989.1 | 10.3 | 33697.7 | 7.6 |
| 9 IM | Indo-Malay | Flooded grasslands and savannas | 27301.5 | 0 | 11335.7 | 41.5 | 10018.1 | 36.7 |
| 9 NT | Neotropic | Flooded grasslands and savannas | 270063.2 | 1 | 54468.2 | 20.2 | 26831.4 | 9.9 |
| 9 PA | Palearctic | Flooded grasslands and savannas | 332514.4 | 0 | 35977.2 | 10.8 | 21479.4 | 6.5 |

| ***Supplementary Table 4. Human Footprint statistics for 2009 within biorealms*** | | | | | |
| --- | --- | --- | --- | --- | --- |
|  |  |  | **Human Footprint Statistics for 2009** | | |
| **Biorealm** | **Realm** | **Biome** | **Mean** | **Standard Deviation** | **Median** |
| 1 AA | Australasia | Tropical and subtropical moist broadleaf forests | 5.8 | 5.0 | 4 |
| 1 AT | Afrotropic | Tropical and subtropical moist broadleaf forests | 6.1 | 4.5 | 5 |
| 1 IM | Indo-Malay | Tropical and subtropical moist broadleaf forests | 11.5 | 7.5 | 10 |
| 1 NA | Nearctic | Tropical and subtropical moist broadleaf forests | 6.8 | 3.8 | 6 |
| 1 NT | Neotropic | Tropical and subtropical moist broadleaf forests | 3.6 | 5.2 | 1 |
| 1 PA | Palearctic | Tropical and subtropical moist broadleaf forests | 12.4 | 5.2 | 13 |
| 10 AA | Australasia | Montane grasslands and savannas | 3.6 | 3.3 | 3 |
| 10 AT | Afrotropic | Montane grasslands and savannas | 9.8 | 5.6 | 8 |
| 10 IM | Indo-Malay | Montane grasslands and savannas | 7.5 | 3.9 | 6 |
| 10 NT | Neotropic | Montane grasslands and savannas | 3.6 | 3.9 | 3 |
| 10 PA | Palearctic | Montane grasslands and savannas | 4.7 | 4.6 | 4 |
| 11 NA | Nearctic | Tundra | 0.1 | 0.8 | 0 |
| 11 PA | Palearctic | Tundra | 0.2 | 1.1 | 0 |
| 12 AA | Australasia | Mediterranean forests woodlands and scrub | 6.1 | 5.5 | 7 |
| 12 AT | Afrotropic | Mediterranean forests woodlands and scrub | 8.7 | 6.7 | 7 |
| 12 NA | Nearctic | Mediterranean forests woodlands and scrub | 10.7 | 11.6 | 5 |
| 12 NT | Neotropic | Mediterranean forests woodlands and scrub | 8.1 | 6.8 | 6 |
| 12 PA | Palearctic | Mediterranean forests woodlands and scrub | 12.5 | 8.6 | 11 |
| 13 AA | Australasia | Deserts and xeric shrublands | 1.7 | 2.6 | 0 |
| 13 AT | Afrotropic | Deserts and xeric shrublands | 5.1 | 4.5 | 4 |
| 13 IM | Indo-Malay | Deserts and xeric shrublands | 15.6 | 7.4 | 15 |
| 13 NA | Nearctic | Deserts and xeric shrublands | 5.2 | 5.7 | 3 |
| 13 NT | Neotropic | Deserts and xeric shrublands | 7.2 | 5.4 | 6 |
| 13 PA | Palearctic | Deserts and xeric shrublands | 3.1 | 4.2 | 2 |
| 14 AA | Australasia | Mangrove | 3.0 | 3.4 | 2 |
| 14 AT | Afrotropic | Mangrove | 8.8 | 6.1 | 7 |
| 14 IM | Indo-Malay | Mangrove | 13.6 | 7.9 | 14 |
| 14 NT | Neotropic | Mangrove | 7.5 | 7.3 | 5 |
| 2 AA | Australasia | Tropical and subtropical dry broadleaf forests | 12.1 | 4.7 | 12 |
| 2 AT | Afrotropic | Tropical and subtropical dry broadleaf forests | 6.1 | 3.3 | 5 |
| 2 IM | Indo-Malay | Tropical and subtropical dry broadleaf forests | 15.5 | 6.5 | 15 |
| 2 NA | Nearctic | Tropical and subtropical dry broadleaf forests | 8.1 | 6.2 | 6 |
| 2 NT | Neotropic | Tropical and subtropical dry broadleaf forests | 7.5 | 5.6 | 7 |
| 3 IM | Indo-Malay | Tropical and subtropical coniferous forests | 13.1 | 5.8 | 14 |
| 3 NA | Nearctic | Tropical and subtropical coniferous forests | 4.5 | 3.4 | 3 |
| 3 NT | Neotropic | Tropical and subtropical coniferous forests | 8.5 | 6.1 | 7 |
| 4 AA | Australasia | Temperate broadleaf and mixed forests | 6.0 | 6.7 | 4 |
| 4 IM | Indo-Malay | Temperate broadleaf and mixed forests | 8.5 | 5.9 | 7 |
| 4 NA | Nearctic | Temperate broadleaf and mixed forests | 10.4 | 10.7 | 7 |
| 4 NT | Neotropic | Temperate broadleaf and mixed forests | 3.4 | 4.5 | 2 |
| 4 PA | Palearctic | Temperate broadleaf and mixed forests | 13.7 | 8.8 | 12 |
| 5 IM | Indo-Malay | Temperate coniferous forests | 9.6 | 7.1 | 7 |
| 5 NA | Nearctic | Temperate coniferous forests | 4.2 | 7.2 | 2 |
| 5 PA | Palearctic | Temperate coniferous forests | 3.7 | 4.7 | 2 |
| 6 NA | Nearctic | Boreal forests taiga | 0.3 | 1.8 | 0 |
| 6 PA | Palearctic | Boreal forests taiga | 1.9 | 4.1 | 0 |
| 7 AA | Australasia | Tropical and subtropical grasslands savannas | 2.5 | 3.1 | 2 |
| 7 AT | Afrotropic | Tropical and subtropical grasslands savannas | 6.3 | 4.4 | 5 |
| 7 IM | Indo-Malay | Tropical and subtropical grasslands savannas | 18.5 | 5.3 | 17 |
| 7 NA | Nearctic | Tropical and subtropical grasslands savannas | 10.9 | 11.0 | 6 |
| 7 NT | Neotropic | Tropical and subtropical grasslands savannas | 5.9 | 5.1 | 5 |
| 7 OC | Oceania | Tropical and subtropical grasslands savannas | 5.1 | 7.4 | 2 |
| 8 AA | Australasia | Temperate grasslands savannas and shrublands | 6.2 | 4.5 | 4 |
| 8 AT | Afrotropic | Temperate grasslands savannas and shrublands | 7.0 | 6.5 | 5 |
| 8 NA | Nearctic | Temperate grasslands savannas and shrublands | 6.5 | 7.6 | 3 |
| 8 NT | Neotropic | Temperate grasslands savannas and shrublands | 6.4 | 5.8 | 4 |
| 8 PA | Palearctic | Temperate grasslands savannas and shrublands | 9.2 | 5.8 | 9 |
| 9 AT | Afrotropic | Flooded grasslands and savannas | 6.1 | 3.6 | 5 |
| 9 IM | Indo-Malay | Flooded grasslands and savannas | 3.6 | 3.8 | 4 |
| 9 NT | Neotropic | Flooded grasslands and savannas | 4.9 | 5.3 | 3 |
| 9 PA | Palearctic | Flooded grasslands and savannas | 10.1 | 10.4 | 6 |
